# High plasma levels of soluble triggering receptor expressed on Myeloid Cells 2 were associated with depression in patients with newly diagnosed type 2 diabetes mellitus

**DOI:** 10.1016/j.jcte.2026.100457

**Published:** 2026-09-09

**Authors:** Eva O. Melin, Mona Landin-Olsson, Magnus Hillman

**Affiliations:** Department of Clinical Sciences, Faculty of Medicine, Lund University, SE-221 84 Lund, Sweden

**Keywords:** anxiety, C-peptide, Depression, Soluble neuropilin-1, Soluble TREM2, Type 2 diabetes

## Abstract

**Aims:**

The hypothesis was that depressed patients compared to non-depressed patients with newly diagnosed type 2 diabetes mellitus (T2DM) had higher levels of soluble Triggering Receptor Expressed on Myeloid Cells 2 (sTREM2) as a sign of increased microglial activation. The aim was to explore a potential association between depression and sTREM2.

**Materials and methods:**

Cross-sectional and multicentre study which included adults with serologically verified, newly diagnosed T2DM. Associations with depression and high sTREM2 (>3.38 pg/mL) were assessed using multiple regression analyses. Potential covariations were explored for age, sex, anxiety, soluble neuropilin-1 (sNRP-1), C-peptide, body mass index (BMI), Haemoglobin A1c, physical inactivity, smoking, and prior cardiovascular disease. ELISA techniques were used to analyse sTREM2, sNRP-1, and C-peptide.

**Results:**

Included were 726 patients (aged 18–94 years, women 38%, depressed 16%, anxious 23%, physically inactive 32%). The prevalence was for high sTREM2 (>3.38 pg/mL) 20%, low sNRP-1 (<225.5 ng/mL) 24%, and high C-peptide (≥1.60 nmol/L) 26%.

Associated with depression (*n* = 630) were anxiety (adjusted odds ratio (AOR) 10.5, *p* < 0.001), low sNRP-1 (AOR 3.3, *p* < 0.001), high sTREM2 (AOR 2.0, *p* = 0.016), high C-peptide (AOR 1.9, *p* = 0.024), and physical inactivity (AOR 1.8, *p* = 0.025).

Associated with high sTREM2 (*n* = 656) were BMI (per kg/m^2^) (AOR 1.08, *p* < 0.001) and depression (AOR 1.8, *p* = 0.015).

**Conclusions:**

Depressed patients compared non-depressed patients with newly diagnosed T2DM had higher levels of sTREM2. High sTREM2, high C-peptide, low sNRP-1, physical inactivity, and anxiety, were independently associated with depression. Depression and BMI were independently associated with high sTREM2. The hypothesis was supported.

## Highlights

• Microglial cells are the major immune cells in the central nervous system.
• Impairment of the functin of microglia can lead to neuroinflammation.
• sTREM2 is a marker of microglial activation.
• High levels of sTREM2 were associated with depression in T2DM patients.

## Graphical abstract

**Figure:**
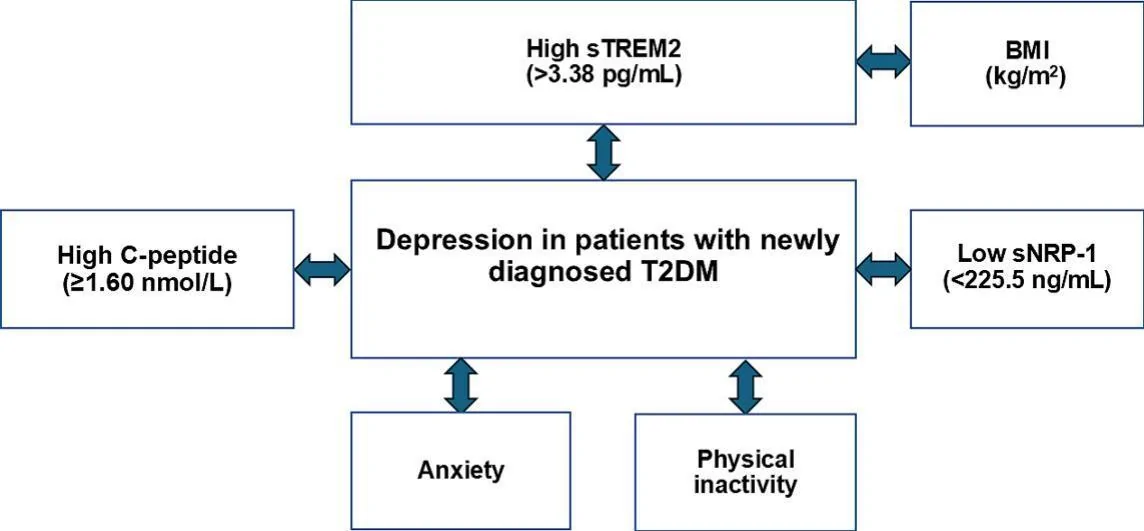

## Introduction

Depression is a risk factor for type 2 diabetes mellitus (T2DM) [1], [2]. Depression is, however, a heterogenous disease with different clinical symptoms and metabolic consequences [3]. Immuno-metabolic depression, which is also named atypical depression, is characterized by increased appetite, low energy levels, systemic low-grade inflammation and metabolic disturbances [3], [4]. Both depression and T2DM are risk factors for dementia [5], [6], and the comorbidity is linked to increased all-cause mortality [2].

Microglial cells are the major immune cells in the central nervous system (CNS) and they are involved in phagocytosis, the release of inflammatory cytokines, regulation of synaptic plasticity, and regulation of the interaction between the immune and nervous systems in response to several types of stressors [7], [8], [9]. Impairment of the normal structure and function of microglia can lead to neuroinflammation and hippocampal degeneration contributing to the development of depression and/or Alzheimer's disease [6], [7], [8], [9], [10], [11]. Microglial activation is mediated by its membrane-bound receptor, the Triggering Receptor Expressed on Myeloid cells 2 (TREM2) [11], [12]. Soluble (s)TREM2 is generated by proteolytic cleavage and shedding of the TREM2 ectodomain or by alternative splicing of TREM2 [12]. The fragment of the receptor, sTREM2, can serve as a marker of microglial activation and can be monitored in plasma or in the cerebrospinal fluid (CSF) [11], [12]. In an experimental study it was demonstrated that the receptor TREM2 promoted adipogenesis [13].

Insulin resistance (IR), which is a main feature of T2DM, is defined as a defect in insulin-mediated control of glucose metabolism [14]. Insulin receptors are expressed throughout the brain [15] and insulin modulates emotional behaviour through a serotonin-dependent process [16]. Brain IR has great impact on the functioning of the brain and contributes to anxiety and depressive behaviour [15], [17], [18], [19], [20], and to global cognitive decline [21]. Increased C-peptide levels and IR have been linked to immuno-metabolic depression [3], [4], Alzheimer's disease [21], [22], cardiovascular disease (CVD) [14], and increased all-cause mortality [23], [24].

We have previously shown that low levels of soluble neuropilin-1 (sNRP-1), anxiety, higher body mass index (BMI), and physical inactivity, were independently associated with depression in this cohort of patients with newly diagnosed T2DM [25].

Despite a well-established relationship between T2DM and depression, the biological characteristics at the time for the diagnosis of T2DM remain insufficiently explored. The hypothesis was that depressed patients with newly diagnosed T2DM had higher levels of sTREM2 as a sign of increased microglial activation. The aim was to explore a potential association between plasma derived sTREM2 and depression in patients with newly diagnosed T2DM.

## Materials and methods

### Participants and study design

This multi-centre and cross-sectional study included adults with newly diagnosed and serologically verified T2DM who had completed the Swedish version of the self-report instrument Hospital Anxiety and Depression Scale (HADS) (*n* = 1027) [25], [26], and had available plasma measurements of sTREM2 (*n* = 726 (71%)). Individuals with gestational diabetes or inability to complete HADS unassisted due to cognitive impairment or inadequate knowledge of Swedish were excluded. Recruitment was conducted over a two-year period starting on the 1st of January 2016. The patients were recruited from five hospitals and 54 primary care units in two Swedish regions, Kalmar and Kronoberg [25], [26]. Clinical data and blood samples were collected within three weeks of the diabetes diagnosis. Logistic regression analyses were performed with depression and high sTREM2 (>3.38 pg/mL) as outcome variables. Comparisons were performed between younger and older participants (<60 *vs* ≥ 60 years), between antidepressant users and non-users, and between the 726 included and the 301excluded patients. Missing data were not imputed; details are presented in Table 1.

**Table 1:** Baseline characteristics and comparisons between 118 depressed and 608 non-depressed patients with newly diagnosed T2DM.

		Baseline characteristics	Depression		
			Yes	No	*P*^*a*^
*n*		726	118	608	
Age (years)		63 (53, 72; 18–94)	61 (51, 72)	63 (54, 72)	0.20 ^b^
Age (years)	<60	312 (43)	57 (48)	255 (42)	0.20
	≥60	414 (57)	61 (52)	353 (58)	
Sex	Women	273 (38)	59 (50)	214 (35)	0.002
	Men	453 (62)	59 (50)	394 (65)	
Depression		118 (16)	–	–	
Anxiety		164 (23)	75 (64)	89 (15)	<0.001
Antidepressants ^c^		75 (18)	28 (35)	14 (14)	<0.001
sTREM2 (pg/mL)		2.0 (1.4, 3.1; 0.4–4682.0)	2.4 (1.5, 3.9)	2.0 (1.4, 2.9)	0.009 ^b^
sTREM2 quintiles (pg/mL)	>3.38	144 (20)	35 (30)	109 (18)	0.006 ^d^
	>2.28 - ≤3.38	144 (20)	26 (22)	118 (19)	
	>1,71 - ≤2.28	148 (20)	19 (16)	129 (21)	
	>1.27 - ≤1,71	144 (20)	18 (15)	126 (21)	
	≤1,27	146 (20)	20 (17)	126 (21)	
High sTREM2 (>3.38 pg/mL)		144 (20)	35 (30)	109 (18)	0.003
Low sNRP-1 (<225.5 ng/mL) ^e^		175 (24)	53 (45)	122 (20)	<0.001
C-peptide (nmol/L)		1.09 (0.77, 1.63; 0.25–5.58)	1.22 (0.86, 1.84)	1.05 (0.76, 1.57)	0.007 ^b^
High C-peptide (≥1.60 nmol/L)		188 (26)	42 (36)	146 (24)	0.009
HbA1c ^f^	(%)	6.7 (6.3, 8.4; 4.9–17.5)	6.7 (6.3, 8.0)	6.7 (6.3, 8.4)	0.58 ^b^
	(mmol/mol)	50 (45, 68; 30–168)	50 (45, 64)	50 (45, 68)	
BMI (kg/m^2^) ^g^		31 (28, 35; 17–55))	32 (28, 37)	31 (28, 35)	<0.025 ^b^
Physical inactivity ^h^		216 (32)	45 (43)	171 (30)	0.009
Smoking ^i^		96 (14)	22 (20)	74 (12)	0.041
Stroke/TIA (prior) ^j^		60 (9)	8 (7)	52 (9)	0.61 ^k^
Myocardial infarction (prior) ^l^		91 (13)	13 (12)	78 (14)	0.52

Data are presented as *n* (%), median (q_1_, q_3;_ min-max) or median (q_1_, q_3_). ^a^ Pearson Chi-Square unless otherwise indicated. ^b^ Mann-Whitney *U* Test. Missing value: ^c^ 312. ^d^ Linear-by-Linear Association. Missing values: ^e^ 5 ^f^ 7; ^g^ 32; ^h^ 51; ^i^ 19; ^j^ 42. ^k^ Fisher's Exact Test. Missing value: ^l^ 44.

### Definition and serological confirmation of type 2 diabetes mellitus

Diabetes mellitus was defined in accordance with the World Health Organisation and American Diabetes Association criteria: *i.e.* fasting glucose, venous or capillary ≥7.0 mmol/L on two different occasions; glucose (venous/capillary) ≥11.1/≥12.2 mmol/L once random or at 120 min during a 75 g oral glucose tolerance test; or Haemoglobin A1c (HbA1c) ≥6.5% (≥48 mmol/mol) [27]. To ensure diagnostic specificity, the diabetes classification was supported by the presence of glutamic acid decarboxylase (GAD) antibody negativity (< 10 units/mL) and C-peptide ≥0.25 nmol/L [28].

### Biochemical analyses

2.3.1 Plasma concentrations of sTREM2 (pg/mL) were measured by enzyme-linked immunosorbent assay (ELISA), using the Human TREM2 DuoSet ELISA kit together with the corresponding ancillary reagent kit (R&D Systems®, Minneapolis, MN, USA). EDTA plasma samples were diluted 1:2 in the assay diluent provided in the ancillary reagent kit and was analysed according to the manufacturer's instructions. Each sample was analysed in duplicate. Standard curves were constructed using a four-parameter logistic (4PL) regression model. Optical density was measured at 450 nm with 580 nm wavelength correction using a FLUOstar OPTIMA microplate reader (BMG LABTECH®, Ortenberg, Germany).

sTREM2 concentrations were subsequently divided into quintiles, with high sTREM2 defined as values within the fifth quintile.

2.3.2 Plasma concentrations of sNRP-1 (ng/mL) were measured by using the commercially available DuoSet ELISA (R&D systems®, Minneapolis, Minnesota, USA). EDTA plasma samples were diluted 1:500 in the assay diluent provided in the ancillary reagent kit and applied to pre-coated 96-well microplates containing capture antibodies against sNRP-1. Plates were incubated for two hours at room temperature. Detection was performed using horseradish peroxidase, generating a colorimetric signal proportional to the sNRP-1 concentration. Absorbance was measured at 450 nm with 580 nm wavelength correction in a FLUOstar OPTIMA ELISA reader (BMG LABTECH®, Ortenberg, Germany). The protein concentration was calculated from a seven-point standard curve using a 4PL model. The intra-assay coefficient of variation was 2.0% [25].

Low sNRP-1 levels were defined as <226 ng/mL corresponding to levels withing the first quartile in 837 patients with available sNRP-1 measurements [25].

Plasma C-peptide concentrations (nmol/L) were analysed by using a commercially available ELISA kit (Mercodia®, article nr 10–1136-01), Uppsala, Sweden) [25], [26]

High C-peptide levels were defined as concentrations ≥1.60 nmol/L, corresponding to the fourth quartile in all 1027 patients.

2.3.4 GAD antibodies (units/mL) were measured by using a commercial ELISA kit from RSR® (Rs-GDE/96, RSR Ltd., Cardiff, UK) [25], [26]. GAD antibody negativity was defined as <10 units/mL [28].

2.3.5 HbA1c (NGSP (%), IFCC (mmol/mol)) was measured for clinical purpose using automated clinical chemistry analysers with high specificity (Olympus AU®, Tokyo, Japan) at local departments of clinical chemistry [25], [26].

### Psychometrically assessed depression and anxiety and the use of antidepressants

Depression and anxiety were defined as ≥8 points on each subscale of HADS (HADS–depression and HADS-anxiety) [25], [26], [29], [30], [31], [32]. The patients were questioned whether they currently used antidepressants. There were two response options, yes or no [25], [26].

### Life-style factors

Physical inactivity was defined as less than 30 min moderate physical activity once a week [25], [26]. Smoking habits were classified as current smokers (daily or occasionally) or non-smokers (not within the last 6 months or never) [25], [26].

### Body measurements

Weight and length were measured by a nurse. BMI (kg/m^2^) was calculated [25], [26].

### Prior cardiovascular disease

Prior stroke/transient ischaemic attack (TIA) and prior myocardial infarction (MI) were registered.

### Statistical analysis

All included continuous variables showed skewed distributions and were therefore presented as median (quartile (q)_1_, q_3_). Group comparisons for continuous data were performed using the Mann-Whitney *U* test. Categorical data were reported as *n* (%) and were analysed by using either Pearson's Chi-Squared test, Fisher's Exact Test or Linear-by-Linear Association. Crude odds ratios (CORs) with 95% confidence intervals (CIs) were calculated for all included variables with depression and high TREM2 (>3.38 pg/mL) as outcome measures. Variables with *p* - values ≤0.10 in the crude analyses were entered into multiple regression models using a Backward Wald procedure and adjusted ORs (AORs) with 95% CI were obtained. Model performance was assessed by using the Omnibus Tests of Model Coefficients and Nagelkerke R Square, and the results were presented below Table 3 and Table 4. A two-sided *p*-value <0.05 was considered statistically significant. All statistical analyses were conducted using SPSS® version 27 (IBM, Chicago, Il, USA).

### Post-hoc power analysis

The prevalence of depression was 24% in 144 patients with high sTREM2 and 14% in 582 patients with low sTREM2 which rendered a power of 80.6% with alpha value 0.05.

## Results

### Baseline characteristics and comparisons between depressed and non-depressed patients

Baseline characteristics for 726 T2DM patients are displayed in Table 1.

Depressed patients (*n* = 118) compared to non-depressed patients (*n* = 608) had higher prevalence of anxiety and low sNRP-1 (<225.5 ng/mL) (both *p* < 0.001); high sTREM2 (>3.38 pg/mL) (*p* = 0.003); high C-peptide (≥1.60 nmol/L) and physical inactivity (both *p* = 0.009); and smoking (*p* = 0.041). They had also higher BMI levels (*p* = 0.025) (Table 1).

### Associations with depression

Independently associated with depression (*n* = 630) were anxiety (AOR 10.5, *p* < 0.001), low sNRP-1 (AOR 3.3, *p* < 0.001), high sTREM2 (AOR 2.0, *p* = 0.016), high C-peptide (AOR 1.9, *p* = 0.024), and physical inactivity (AOR 1.8, *p* = 0.025) (Table 2).

**Table 2:** Associations with depression in patients with newly diagnosed T2DM.

		Depression			
		COR (CI 95%)	*P*	AOR (CI 95%)	*P*^a^
Age (per year)		0.99 (0.97–1.00)	<0.11	–	–
Sex (women)		1.8 (1.2–2.7)	0.003	1.3 (0.8–2.2)	0.26
Anxiety		10.2 (6.6–15.7)	<0.001	10.5 (6.3–17.4)	<0.001
Antidepressants		3.3 (1.9–5.7)	<0.001	–	–
sTREM2 quintiles (pg/mL)	>3.38	2.0 (1.1–3.7)	0.023	–	–
	>2.28 – ≤3.38	1.4 (0.7–2.6)	0.31	–	–
	>1,71 – ≤2.28	0.9 (0.5–1.8)	0.83	–	–
	>1.27 – ≤1,71	0.9 (0.46–1.8)	0.76	–	–
	≤1,27	1		–	–
High sTREM2 (≥1.60 nmol/L)		1.9 (1.2–3.0)	0.004	2.0 (1.1–3.5)	<0.016
Low sNeuropilin-1 (<225.5 ng/mL)		3.2 (2.1–4.9)	<0.001	3.3 (2.0–5.5)	<0.001
High C-peptide (≥1.60 nmol/L)		1.7 (1.1–2.7)	0.009	1.9 (1.1–3.2)	0.024
HbA1c	(per %)	1.0 (0.9–1.1)	0.43	–	–
	(per mmol/mol)	1.00 (0.99–1.01)		–	–
BMI (per kg/m^2^)		1.05 (1.01–1.08)	<0.006	1.02 (0.97–1.06)	0.44
Physical inactivity		1.8 (1.1–2.7)	0.010	1.8 (1.1–3.0)	0.025
Smoking		1.7 (1.0–2.9)	0.043	1.3 (0.7–2.5)	0.41
Stroke/TIA (prior)		0.8 (0.4–1.8)	0.61	–	–
Myocardial infarction (prior)		1.1 (0.6–2.1)	0.69	–	–

a Logistic regression (Backward: Wald): *n* = 630; Omnibus Tests of Model Coefficients *p* < 0.001; Nagelkerke R^2^ 0.336.

### Comparisons between patients with high and low sTREM2

Patients with high sTREM2 (*n* = 144) compared to patients with lower levels of sTREM2 (*n* = 582) had higher median levels of C-peptide (nmol/L) and BMI (kg/m^2^) (both *p* < 0.001) (Table 3). They also had higher prevalence of depression (*p* = 0.003) and prior stroke/TIA (*p* = 0.024) and were more likely to be women (*p* = 0.023).

**Table 3:** Comparisons between 144 T2DM patients with high sTREM2 and 582 T2DM patients with lower sTREM2.

		High sTREM2 (>3.38 pg/mL)	Low sTREM2 (≤3.38 pg/mL)	*P*^*a*^
*n*		144	582	
Age (years)		64 (54, 73)	63 (53, 71)	0.50 ^b^
Age (years)	<60	54 (38)	258 (44)	0.14
	≥60	90 (62)	324 (56)	
Sex	Women	66 (46)	207 (36)	0.023
	Men	78 (54)	375 (64)	
Depression		35 (24)	83 (14)	0.003
Anxiety		36 (25)	128 (22)	0.44
Antidepressants		18 (20)	57 (18)	0.56
Low sNeuropilin-1 (<225.5 ng/mL)		32 (22)	143 (25)	0.52
C-peptide (nmol/L)		1.30 (0.90, 1,74)	1.03 (0.73, 1.57)	<0.001 ^b^
HbA1c	(%)	6.9 (6.5, 0.1)	6.7 (6.3, 8.1)	0.076 ^b^
	(mmol/mol)	52 (47, 76)	50 (45, 65)	
BMI (kg/m^2^)		33 (29, 37)	31 (28, 34)	<0.001^b^
Physical inactivity		48 (35)	168 (31)	0.36
Smoking		20 (14)	76 (13)	0.82
Stroke/TIA (prior)		19 (14)	41 (8)	0.024
Myocardial infarction (prior)		17 (12)	74 (14)	0.72

Data are presented as *n* (%) or as median (q_1_, q_3_). ^a^ Pearson Chi-Square unless otherwise indicated. ^b^ Mann-Whitney *U* Test.

### Associations with high sTREM2

Independently associated with high sTREM2 (*n* = 656) were BMI (per kg/m^2^) (AOR 1.08, *p* < 0.001) and depression (AOR 1.8, *p* = 0.015) (Table 4).

**Table 4:** Associations with high sTREM2 in patients with newly diagnosed T2DM.^a^

	High sTREM2 (>3.38 pg/mL)			
	COR (95% CI)	*P*	AOR (95% CI)	*P*^a^
Age (per year)	1.00 (0.99–1.02)	0.76	–	–
Women	1.5 (1.1–2-2)	0.023	1.3 (0.9–1.9)	0.23
Depression	1.9 (1.2–3.0)	0.004	1.8 (1.1–2.9)	0.015
Anxiety	1.2 (0.8–1.8)	0.44	–	–
Antidepressants	1.2 (0.7–2.2)	0.56	–	–
Low sNeuropilin-1 (<225.5 ng/mL)	0.9 (0.6–1.3)	0.52	–	–
C-peptide (per nmol/L)	1.4 (1.1–1.8)	0.003	1.2 (0.9–1.6)	0.14
High C-peptide (≥1.60 nmol/L)	1.6 (1.1–2.3)	0.024	–	–
HbA1c (per mmol/mol)	1.00 (1.00–1.01)	0.31	–	–
BMI (per kg/m^2^)	1.09 (1.05–1.12)	<0.001	1.08 (1.05–1.12)	<0.001
Physical inactivity	1.2 (0.8–1.8)	0.36	–	–
Smoking	1.1 (0.6–1.8)	0.81	–	–
Stroke/TIA (prior)	1.9 (1.1–3.4)	0.026	1.8 (1.0–3.3)	0.062
Myocardial infarction (prior)	0.9 (0.5–1.6)	0.72	–	–

a Multiple logistic regression (Backward: Wald): *n* = 656; Omnibus Tests of Model Coefficients <0.001; Nagelkerke R^2^ 0.080.

### Comparisons between younger and older patients

Comparisons between 312 younger patients (<60 years) and 414 older patients (≥60 years) showed that the prevalence did not differ for high sTREM2 (17% *vs* 22%, *p* = 0.14) or low sNRP-1 (27% *vs* 22%, *p* = 0.10). The prevalence of high C-peptide was lower in the younger patients than in the older patients (20% *vs* 30%, *p* = 0.001).

### Comparisons between users and non-users of antidepressants

Comparisons between 75 users and 339 non-users of antidepressants showed that the prevalence did not differ for high sTREM2 (24% *vs* 21%, *p* = 0.56), high C-peptide (29% *vs* 27%, *p* = 0.62), low sNRP-1 (*n* = 410) (33% *vs* 23%, *p* = 0.053), prior MI (*n* = 405) (19% *vs* 12%, *p* = 0.15), or stroke/TIA (*n* = 405) (9% *vs* 9%, *p* = 0.98).

### Comparisons between included and excluded patients

Comparisons between the 726 included and the 301 excluded patients without available sTREM2 showed that the included patients were older and had higher prevalence of depression, anxiety, prior MI, prior stroke/TIA, and lower levels of BMI and HbA1c (all *p* < 0.05). The prevalence of low sNRP-1, high C-peptide, physical inactivity and smoking did not differ between the included and excluded patients (all *p* ≥ 0.05).

## Discussion

In this cross-sectional study of 726 adults with newly diagnosed and serum-verified T2DM, the main finding was that the depressed patients compared to the non-depressed patients had higher levels of sTREM2. High sTREM2 (>3.38 pg/mL), high C-peptide (≥1.60 nmol/L), low sNRP-1 (<225.5 ng/mL), physical inactivity, and anxiety, were independently associated with depression. Depression and BMI were independently associated with high sTREM2.

To our knowledge, we are the first to demonstrate significantly higher levels of plasma sTREM2 in depressed patients compared to in non-depressed patients with newly diagnosed T2DM. Furthermore, we are the first to demonstrate that high sTREM2, high C-peptide, low sNRP-1, physical inactivity, and anxiety, were independently associated with depression. The demonstrated independent associations between depression, BMI and high sTREM2 are also, to our knowledge, new findings. The hypothesis was supported. Previous research has shown that microglial activation plays a very important role in the pathogenesis of depression [6], [7], [9], [10], [33] and that sTREM2 can serve as a marker of microglial activation [11], [12]. However, in this study increased microglial activation in depressed patients was not verified by any other measurements or techniques. The demonstrated association between BMI and high sTREM2 is in line with a previous experimental study showing that the receptor TREM2 promoted adipogenesis [13].

The association between high C-peptide and depression is in line with findings of IR in non-diabetic patients with immuno-metabolic depression [3], [4]. The finding is important as previous research has shown that IR has great impact on the brain, including on emotional behaviour, executive and global cognitive functions, as well as the development of depression and Alzheimer's disease [3], [15], [16], [17], [18], [20], [21], [22], [34].

Altogether, in these patients with newly diagnosed T2DM we found three biomarkers associated with depression which previously have been linked to Alzheimer's disease, *i.e.* sTREM2, c-peptide and sNRP-1 [[6], [21], [22], [35]].

The depressed patients with newly diagnosed T2D had a very high prevalence of anxiety compared to the non-depressed. Depression in combination with anxiety is a particularly high risk factor for the development of T2DM [1]. There was also a clear association between physical inactivity and depression. Physical inactivity is an established risk factor for depression [36], but may also be a symptom of low energy and leaden paralysis typical for immuno-metabolic depression [3].

There were several strengths of our study. T2DM was serologically verified. Precise ELISA techniques were used for sTREM2, sNRP-1, C-peptide and GAD antibodies. We controlled for relevant variables such as sex [30], anxiety [1], BMI [3], [31], physical inactivity [[3], [30], [36]], smoking [30], sNRP-1 [25], HbA1c [32], and cardiovascular disease [[30], [37]], which all have been linked to depression. Each multiple logistic regression model was evaluated by the Omnibus Tests of Model Coefficients and by Nagelkerke R Square. The post-hoc power calculation showed an adequate power of 80.6% which confirms that an adequate number of participants were included. According to previous studies, MI [30] and stroke [[37], [38]] are associated with increased prevalence of depression, which was not demonstrated in our study. Therefore, we explored whether patients with prior MI or stroke/TIA to a higher degree used antidepressants, which they did not.

The first limitation of the study was that microglial activation was not explored and confirmed by a neuroimaging technique [39]. Another limitation was that we do not know if patients with Alzheimer's disease were included in the study. In western Europe, Alzheimer's disease is very rare in persons younger than 60 years [40]. Therefore, we performed sub analyses that showed that the prevalence of high sTREM2 and low sNRP-1 did not differ between younger patients (<60 years) and older patients. The prevalence of high C-peptide was higher in the older patients which is in accordance with previous research showing that IR increases with age [41]. A third limitation was that psychometrically assessed depression was not clinically confirmed, but HADS-D has shown high validity for assessing depressive symptoms and has been widely used in research [25], [26], [29], [30], [31], [32]. Further, the association between the use of antidepressants and depression was very strong, which supports that HADS-D is clinically relevant. A fourth limitation was that the generalizability was affected by several differences between included patients and excluded patients. For example, the prevalence of depression was 4% higher in patients with performed TREM2 biochemical analyses compared to the prevalence in the whole cohort of 1027 patients (12%) [25], [26]. A fifth limitation was the reduced number of respondents to the question regarding current use of antidepressants.

In further research of depression, we suggest that in addition to measuring plasma sTREM2 it would be of value to use magnetic resonance imaging (MRI) for exploration and verification of potential microglial activation [39]. It would also be of interest to explore whether sTREM2, sNRP-1 and C-peptide can be used for supporting a diagnosis of depression and for evaluation of treatment efficacy in depressed patients. GLP-1 receptor agonists (GLP-1RAs) directly target IR [42] and have the potential to reduce depressive symptoms [43]. Whether GLP-1Ras reduce microglial activation in depressed patients is another subject for further exploration. It would also be of interest to explore whether increased awareness and identification of immuno-metabolic depression in people without T2DM and subsequent treatment specifically targeting IR, would result in lower incidence of T2DM and increased rates of recovery from depression.

Clinically, we suggest that all patients with newly diagnosed T2DM should be examined for depression, and in case of positive findings, the patients should acquire individualized treatment. Certain classes of antidepressants have shown effects on microglial activation, but not all classes [33]. Due to the deleterious effects of IR on brain function [3], [15], [16], [17], [18], [20], [21], [34], we recommend that all patients with depression and increased C-peptide levels should have treatment targeting IR, including encouragement of increased physical activity, weight reduction and medication [3].

## Conclusion

In this cohort of patients with newly diagnosed T2DM, the depressed patients compared to the non-depressed patients had higher levels of sTREM2. High sTREM2, high C-peptide, low sNRP-1, physical inactivity, and anxiety, were independently associated with depression. Depression and BMI were independently associated with high sTREM2. The hypothesis was supported.

## Artificial intelligence

We did not use artificial intelligence for any purpose.

## Funding

This research was supported by the Research Council of Southeastern Sweden (FORSS) Linkoping, Sweden, grant numbers: FORSS-845251, FORSS-940428, FORSS-968754. The funding sources were not involved in the collection, analysis or interpretation of the data, in the writing of the report, or in the decision to submit the article for publication.

## CRediT authorship contribution statement

**Eva O. Melin:** Writing – review & editing, Writing – original draft, Resources, Methodology, Investigation, Funding acquisition, Formal analysis, Data curation, Conceptualization. **Mona Landin-Olsson:** Writing – review & editing, Resources, Methodology, Formal analysis, Conceptualization. **Magnus Hillman:** Writing – review & editing, Writing – original draft, Resources, Methodology, Formal analysis, Data curation, Conceptualization.

## Ethics statement

The study was approved by the Regional Ethical Review Board of Linköping University, Linköping (Registration nos. 2015/350–31, date 2016-01-12, and 2017/354–32, date 2017-08-18). All participants provided written informed consent. The research was performed in accordance with the Declaration of Helsinki.

## Declaration of competing interest

The authors declare that they have no known competing financial interests or personal relationships that could have appeared to influence the work reported in this paper.
